# Circulating Piezo 1 Levels in Complex Regional Pain Syndrome Type 1 and Their Association with Time from Neridronate Treatment

**DOI:** 10.3390/biomedicines14061266

**Published:** 2026-06-01

**Authors:** Elisa Assirelli, Jacopo Ciaffi, Susanna Naldi, Francesco Ursini, Simona Neri

**Affiliations:** 1 Medicine and Rheumatology Unit, IRCCS Istituto Ortopedico Rizzoli, 40136 Bologna, Italy; elisa.assirelli@ior.it (E.A.); jacopo.ciaffi@ior.it (J.C.); susanna.naldi@ior.it (S.N.); francesco.ursini@ior.it (F.U.); 2 Department of Biomedical and Neuromotor Sciences (DIBINEM), Alma Mater Studiorum University of Bologna, 40136 Bologna, Italy

**Keywords:** CRPS-1, Piezo 1, biomarkers, neridronate

## Abstract

**Background**: Complex Regional Pain Syndrome type 1 (CRPS-1) is a multifactorial disorder characterized by persistent pain, neuroinflammation, and tissue remodeling following trauma in the absence of overt nerve injury. Despite advances in understanding its pathophysiology, the mechanisms underlying the transition to chronic pain remain incompletely defined, and reliable circulating biomarkers are lacking. Piezo-type mechanosensitive ion channel component 1 (Piezo 1), a mechanosensitive ion channel that transduces mechanical stimuli into intracellular calcium signaling, has emerged as a regulator of inflammation, extracellular matrix remodeling, and cellular stress responses. Experimental evidence indicates that Piezo 1 activation can modulate cytokine production and mechanotransduction pathways relevant to chronic pain and inflammatory conditions. **Methods**: In this study, we evaluated circulating Piezo 1 levels in CRPS-1 patients and explored their association with clinical parameters and response to neridronate treatment. **Results**: Although Piezo 1 levels were significantly altered compared to controls, no associations were observed with pain intensity or therapeutic response. **Conclusions**: These findings suggest that, despite its biological relevance, circulating Piezo 1 is not a clinically informative biomarker in CRPS-1. The results support a predominantly local role of Piezo 1-mediated mechanotransduction in processes relevant to chronic inflammation and nociceptive sensitization.

## 1. Introduction

Complex Regional Pain Syndrome type 1 (CRPS-1) is a chronic and disabling condition characterized by persistent pain disproportionate to the inciting event and associated with sensory, vasomotor, sudomotor, and trophic abnormalities [1,2]. The syndrome typically develops following trauma and evolves through a multifactorial process involving neurogenic inflammation, immune dysregulation, altered sympathetic activity, and central sensitization [3,4,5]. A defining feature of CRPS-1 is the persistence of chronic pain beyond normal tissue healing. This phenomenon is sustained by inflammatory mediators, altered nociceptive signaling, and maladaptive neuroplasticity. Increased levels of cytokines and chemokines contribute to peripheral sensitization, while central nervous system alterations, including cortical reorganization, further reinforce chronic pain states [4,6,7,8,9]. Chronic pain conditions are also frequently associated with impaired sleep quality, and growing evidence suggests a bidirectional relationship between sleep disturbances, pain perception, and psycho-emotional dysregulation, potentially contributing to disease persistence and symptom amplification [10]. Mechanical factors are increasingly recognized as contributors to CRPS pathogenesis. Tissue injury leads to edema, fibrosis, and extracellular matrix (ECM) remodeling, resulting in altered tissue stiffness and persistent mechanical stress [11,12]. These biomechanical changes can act as continuous stimuli capable of sustaining inflammation and nociceptive activation. In this context, the ECM plays a dynamic role in regulating cellular responses, including inflammation and nerve regeneration, and its dysregulation has been linked to neuropathic pain [12,13]. Mechanotransduction, defined as the conversion of mechanical stimuli into biochemical signals, has therefore emerged as a key regulatory mechanism in chronic pain conditions. Among mechanosensitive elements, the ion channel Piezo 1 has gained increasing attention due to its ability to translate mechanical forces into intracellular calcium signaling [14,15].

Piezo 1 is widely expressed in musculoskeletal tissues, endothelial cells, and immune cells, where it regulates cellular responses to mechanical stress [15,16,17]. Although PIEZO1 is a membrane-associated mechanosensitive ion channel, circulating forms or fragments potentially released during cellular stress, inflammation, tissue remodeling, or extracellular vesicle turnover may reflect ongoing pathological processes and therefore represent a biologically plausible exploratory biomarker. Activation of Piezo 1 induces calcium influx and triggers downstream signaling pathways, including NF-κB, MAPK, and inflammasome activation, linking mechanical stimuli to inflammatory responses [16,17,18,19]. In addition, Piezo 1 has been implicated in extracellular matrix remodeling, fibrosis, and cellular stress responses [17,18,19,20].

Recent studies have also highlighted the role of Piezo 1 in immune regulation and inflammatory signaling, further supporting its involvement in pathological processes characterized by chronic inflammation [19,20,21,22]. Moreover, mechanosensitive pathways have been implicated in nociceptive processing and neuropathic pain, suggesting a potential role of Piezo 1 in chronic pain conditions [12,13].

Other nociceptive ion channels, including transient receptor potential vanilloid 1 (TRPV1) and acid-sensing ion channels (ASICs), have also been implicated in inflammatory and neuropathic pain mechanisms [23,24,25], suggesting that CRPS-1 pain sensitization likely involves a broader network of mechanosensitive and nociceptive pathways.

Despite increasing evidence implicating Piezo 1 in mechanotransduction, inflammation, fibrosis, and nociceptive signaling, its potential involvement in CRPS-1 has not yet been investigated. In particular, no previous studies have evaluated circulating Piezo 1 levels in CRPS-1 patients or explored their possible association with chronic pain severity and therapeutic response. This represents an important gap in the current understanding of mechanosensitive pathways in CRPS-1 and limits the identification of potential biomarkers related to disease persistence and tissue remodeling.

Given the central role of mechanical stress, inflammation, and chronic pain in CRPS-1, Piezo 1 represents a biologically plausible mediator linking these processes. The aim of this study was therefore to evaluate serum Piezo 1 levels in CRPS-1 patients and to investigate their association with clinical parameters and response to treatment.

## 2. Materials and Methods

### 2.1. Patients and Samples

CRPS-1 patients and control samples were obtained from the Rheumatology Biobank of the Istituto Ortopedico Rizzoli, Bologna, Italy [26]. Patient inclusion criteria: diagnosis of CRPS-1, treatment with neridronate within 3 months from algodystrophy onset, and availability of suitable serum samples. No exclusion criteria were applied. Controls were patients attending the rheumatology outpatient clinic with any known osteometabolic disease (osteoporosis, Paget’s disease, or previous algodystrophy) or any prior exposure to bisphosphonates.

Peripheral blood samples of 37 CRPS-1 patients (mean age 63.7 ± 13.6 years) and 22 healthy controls (mean age 55.2 ± 15.4 years). Six out of the 37 CRPS-1 patients were excluded due to the absence of serum samples suitable for analysis, leaving 31 patients. Among the final study population, 31 CRPS-1 patients included 21 females and 10 males, while the 22 healthy controls included 10 females and 12 males. The mean time from neridronate treatment in the CRPS-1 cohort was 18.2 ± 20.7 months. All patients participating in the Biobank project provided written informed consent, and the study was approved by the CE-AVEC Ethical Committee (protocol N. 206/2023/Sper/IOR). For each patient, serum was obtained from peripheral blood with standard procedures [26]. A subset of patients (*n* = 10) with paired pre- (T0) and post-treatment (T1) samples was identified for exploratory longitudinal analysis. The total number of serum samples, therefore, exceeded the number of individual patients.

Patients diagnosed with CRPS-1 were included according to clinical diagnostic criteria; all patients fulfilled the Budapest diagnostic criteria for CRPS-1 at the time of diagnosis [27]. Clinical characterization of the CRPS-1 cohort included affected anatomical site, comorbidities, and concomitant medications. Due to the retrospective nature of the study and the heterogeneous timing of serum collection after neridronate treatment, disease duration and CRPS stage at the time of blood sampling were not consistently available for all patients. CRPS is defined by continuing regional pain disproportionate to any inciting event and accompanied by sensory, vasomotor, sudomotor, or trophic abnormalities.

All patients received intravenous neridronate therapy according to established treatment protocols for CRPS (100 mg intravenously, administered four times, each dose given 3 days apart) [28].

Intravenous neridronate has been demonstrated to be an effective treatment for CRPS-1, improving pain and functional outcomes through modulation of bone metabolism and inflammatory processes. Long-term follow-up studies have confirmed its sustained efficacy and safety in this condition [29,30]. Clinical variables included:

- Months from neridronate treatment.
- Self-perceived health status, assessed using the EQ-5D visual analogue scale (EQ-5D VAS).
- Treatment response.

Treatment response was evaluated at the last available follow-up visit according to patient-reported clinical outcomes routinely collected in clinical practice. Patients were classified as responders or non-responders according to criteria previously adopted in our real-life study on intravenous neridronate treatment in CRPS-1 [30]. Briefly, complete responders were defined as patients showing both: (i) a clinically meaningful reduction in pain intensity exceeding the minimal clinically important difference (MCID) for pain scales, corresponding to a relative reduction ≥50% and an absolute reduction ≥3 points in the VAS/NPRS score; and (ii) an improvement of at least 5 points in at least five PROMIS-29 domains, as previously described [30]. Clinical response classification was performed independently of Piezo 1 measurements, which were not available to clinicians during routine patient evaluation.

Patients received concomitant treatments according to their individual comorbidities and standard clinical management. In addition, all patients received calcium and vitamin D supplementation according to the institutional treatment protocol for intravenous neridronate therapy.

### 2.2. Measurement of Piezo 1 and Statistical Analysis

Serum Piezo 1 levels were quantified using a commercially available sandwich ELISA kit (FineTest^®^, EH15116, Wuhan Fine Biotech Co., Wuhan, China) according to the manufacturer’s instructions. The assay detection range was 0.313–20 ng/mL, with a reported sensitivity of 0.188 ng/mL and intra- and inter-assay coefficients of variation below 6%. Serum samples were diluted 1:2 prior to analysis, and final concentrations were corrected according to the dilution factor. Selected samples were analyzed in duplicate to verify assay reproducibility. Optical density was measured at 450 nm and concentrations were calculated using a standard curve.

Data distribution was evaluated using descriptive statistics. Data were expressed as medians, interquartile ranges, minimum and maximum values; means ± standard deviation (SD), as appropriate. Group comparisons were performed using the Mann–Whitney U test. Paired comparisons between pre- and post-treatment samples were performed using the Wilcoxon signed-rank test or paired *t*-test, depending on data distribution. Correlations between Piezo 1 levels and clinical variables were assessed using Spearman correlation analysis. A *p*-value < 0.05 was considered statistically significant. Data were analyzed and graphed using the GraphPad Prism software version 9.0 (GRAPHPAD SOFTWARE, La Jolla, CA, USA).

The STROBE checklist and flowchart of the studyare provided in the Supplementary Materials.

## 3. Results

### 3.1. Study Population

The study included a total of 63 subjects, comprising 31 samples from CRPS-1 patients and 22 healthy controls. Within the CRPS cohort, samples were collected at multiple time points relative to neridronate treatment (0 to 48 months), allowing exploratory assessment of temporal trends. In a subgroup of 10 patients, paired pre- (T0) and post-treatment (T1) serum samples were available for exploratory longitudinal analyses.

The CRPS cohort showed a higher proportion of female patients, consistent with the known epidemiology of the disease.

Demographic and clinical characteristics are summarized in Table 1.

### 3.2. Circulating Piezo 1 Levels in CRPS-1 and Controls

**Figure 1:**
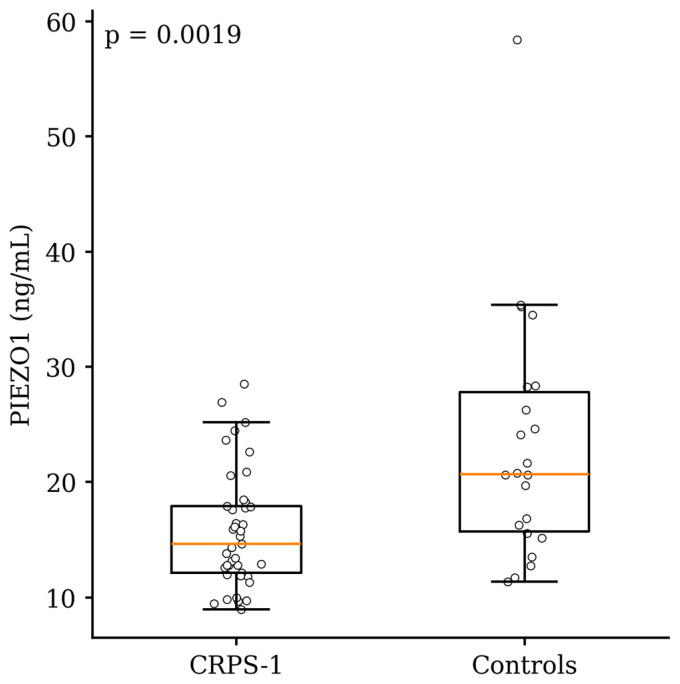
**Serum Piezo 1 level in CRPS-1 patients and healthy controls.** Box plot showing circulating Piezo 1 concentration measured by ELISA in patients with complex regional pain syndrome type 1 (CRPS-1) and healthy controls. The white circles represent individual observations. Box plots represent the median (orange line) and interquartile range (IQR), with whiskers indicating the minimum and maximum values. Piezo 1 levels were significantly lower in CRPS-1 patients compared with controls (Mann–Whitney U test, *p* = 0.0019).

Serum Piezo 1 concentration (ng/mL) was significantly lower in CRPS-1 patients compared with healthy controls. CRPS-1: mean 15.77, median 14.64, controls: mean 23.26, median 20.69 (Mann–Whitney U test: *p* = 0.0019). This finding indicates a clear difference in circulating Piezo 1 level between CRPS-1 patients and non-affected individuals (Figure 1).

### 3.3. Longitudinal Analysis of Piezo 1 Levels Before and After Treatment

Paired serum samples at T0 (pre-treatment) and T1 (post-treatment) were compared within a subgroup of CRPS-1 patients, showing that circulating Piezo 1 levels did not significantly change following treatment (T0 vs. T1: 16.89 ± 5.48 vs. 15.86 ± 4.77 ng/mL, *p* = 0.625). Individual trajectories demonstrated heterogeneous patterns, with no consistent trend across patients. The mean variation in Piezo 1 levels (ΔPiezo 1) was −1.03 ng/mL, indicating no systematic modulation over time. Overall, these findings suggest that circulating Piezo 1 levels remain relatively stable and are not significantly influenced by treatment in CRPS-1 patients.

### 3.4. Association Between Piezo 1 and Time from Neridronate Treatment

**Figure 2:**
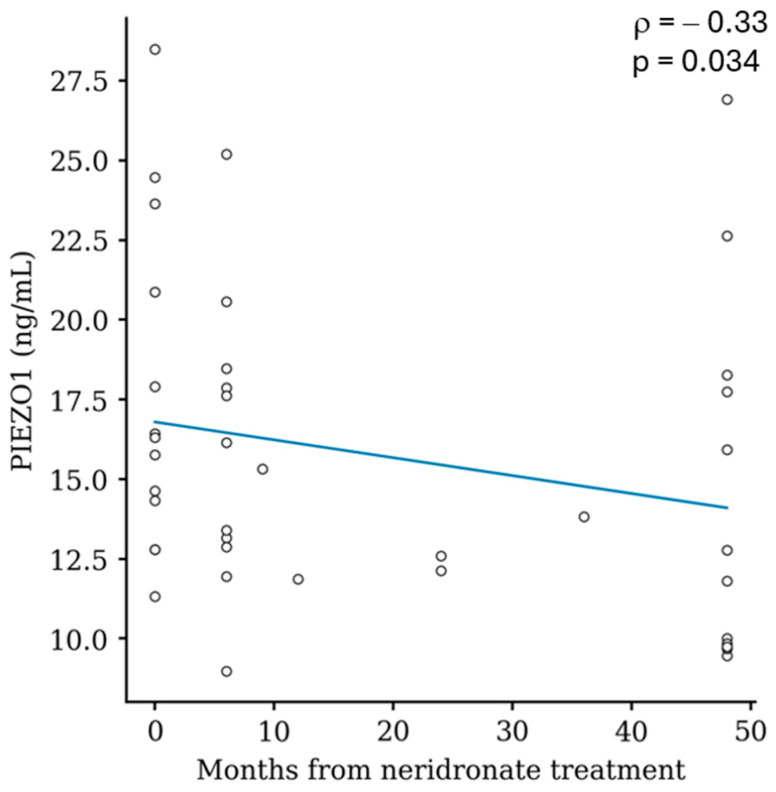
**Correlation between serum Piezo 1 levels and time from neridronate treatment.** Scatter plot showing the relationship between circulating Piezo 1 concentrations and months from neridronate treatment in CRPS-1 patients. The solid line represents the linear regression fit and is included for visualization purposes only. The Spearman correlation coefficient (ρ) and corresponding *p*-value are displayed within the plot. A modest inverse correlation was observed (ρ = −0.33, *p* = 0.034).

Within the CRPS-1 cohort, circulating Piezo 1 levels showed a modest but statistically significant inverse correlation with time from treatment (Spearman ρ = −0.33, *p* = 0.034). This finding indicates an exploratory inverse association between circulating Piezo 1 levels and elapsed time since neridronate treatment. However, given the predominantly cross-sectional nature of the analysis, this association may also reflect differences in disease duration, recovery stage or other clinical variables (Figure 2).

### 3.5. Relationship Between Piezo 1, Self-Perceived Health Status and Treatment Response

No significant correlation was observed between serum Piezo 1 levels and self-perceived health status measured by EQ-5D VAS (Spearman ρ = −0.015, *p* = 0.928).

**Figure 3:**
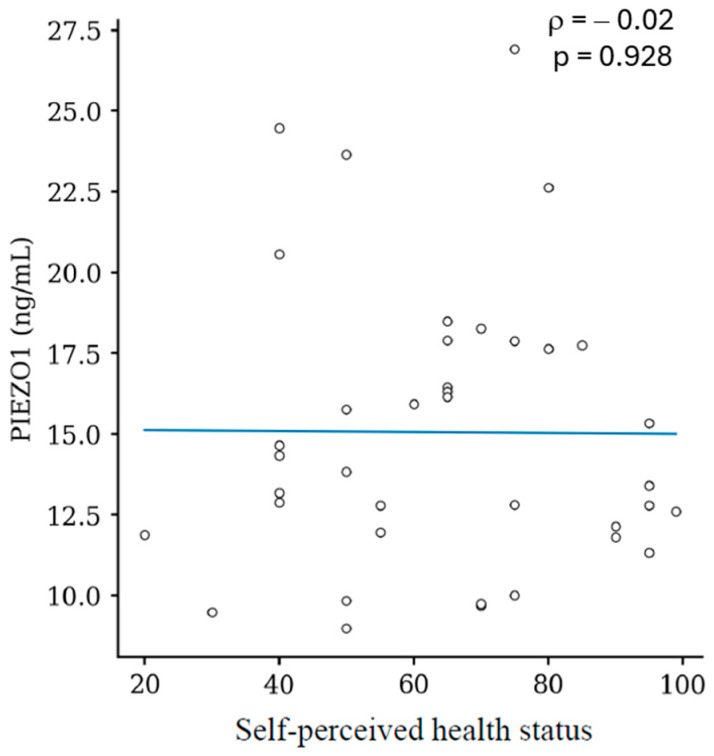
**Correlation between serum Piezo 1 levels and self-perceived health status.** The scatter plot illustrates the relationship between circulating Piezo 1 concentrations and self-perceived health status measured using the EQ-5D visual analogue scale (VAS). The solid line represents the linear regression fit. The Spearman correlation coefficient (ρ) and *p*-value are shown within the plot. No significant correlation was observed (ρ = −0.015, *p* = 0.928).

These results indicate that circulating Piezo 1 does not reflect subjective pain severity in CRPS-1 patients (Figure 3).

**Figure 4:**
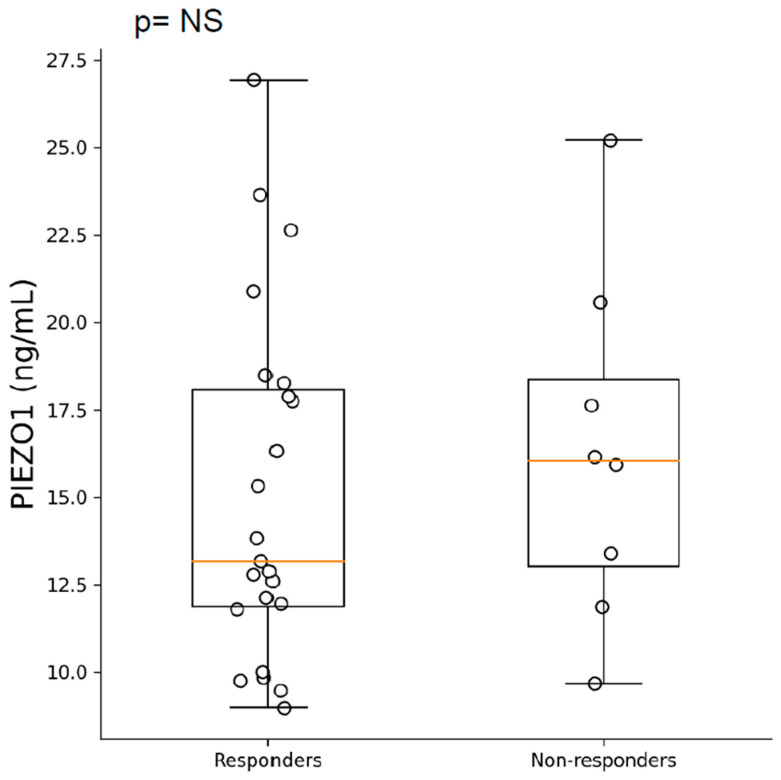
**Serum Piezo 1 levels according to treatment response.** Box plot comparing circulating Piezo 1 concentration between responders (n = 23) and non-responders (n = 8) to neridronate therapy in CRPS-1 patients. The white circles represent individual observations. The central orange line represents the median, the box indicates the interquartile range (IQR), and whiskers represent the minimum and maximum values. No significant differences were observed between the two groups (Mann–Whitney U test, *p* = 0.519).

Patients were classified as responders or non-responders based on clinical criteria. No significant differences in Piezo 1 levels were observed between the two groups. Responders: mean 15.37 ng/mL, non-responders: mean 16.75 ng/mL (*p* = 0.519). This finding suggests that circulating Piezo 1 levels are not associated with clinical response to neridronate treatment (Figure 4).

### 3.6. Exploratory Correlation Analysis with Clinical and Laboratory Variables

Exploratory analyses were performed to assess associations between Piezo 1 levels and laboratory parameters. Significant correlations included: TSH (mUi/mL; 9/31): positive correlation (ρ = 0.764, *p* = 0.002); Eosinophils (%; 17/31): positive correlation (ρ = 0.573, *p* = 0.010); Monocytes (%; 17/31): positive correlation (ρ = 0.509, *p* = 0.026). Exploratory descriptive stratification according to time from treatment was also performed using the cohort median (12 months) as a cutoff. Patients sampled within 12 months from treatment showed numerically higher circulating Piezo 1 levels compared with those sampled after longer follow-up periods, close to statistical significance (*p* = 0.050n). Given the number of comparisons and limited sample size, these findings should be considered hypothesis-generating only.

## 4. Discussion

The present study evaluated circulating Piezo 1 levels in CRPS-1 patients, showing significantly altered serum levels compared with controls but no association with clinical severity or treatment response. These findings highlight the complexity of translating mechanotransductive pathways into clinically useful circulating biomarkers in chronic pain conditions. Previous experimental studies have already implicated Piezo 1 in mechanotransduction, inflammatory signaling, extracellular matrix remodeling, and nociceptive sensitization [14,15,16,17,18,19,20,21]. In CRPS-1, persistent tissue injury and altered biomechanical environments may contribute to abnormal mechanosensitive signaling through interactions between mechanical stress, neuroinflammation, and extracellular matrix remodeling [11,12,13]. Increased tissue stiffness and inflammatory activation may further sustain Piezo 1-mediated calcium-dependent signaling and local immune responses [17,18,19,20]. Despite this biological rationale, our results indicate that circulating Piezo 1 levels do not correlate with clinical parameters. Inflammatory mediators, including cytokines and chemokines, play a central role in amplifying nociceptive signaling and contributing to disease persistence [6,7]. Within this framework, mechanotransduction represents an important dimension of CRPS pathophysiology. Tissue injury induces structural and biomechanical alterations, including edema, fibrosis, and extracellular matrix remodeling, resulting in persistent mechanical stress [11,12]. The ECM is increasingly recognized as an active regulator of cellular behavior, capable of modulating inflammation and nerve regeneration [12,13]. Alterations in ECM stiffness and composition have been directly linked to neuropathic pain and impaired tissue repair. Piezo 1 is a central mediator of mechanotransduction and plays a key role in converting mechanical stimuli into intracellular calcium signaling [14,15]. Activation of Piezo 1 induces calcium influx and triggers downstream pathways involved in inflammation, including NF-κB activation, cytokine production, and inflammasome signaling [16,17,18,19]. Experimental evidence indicates that Piezo 1 activation can promote the release of pro-inflammatory mediators and contribute to a pro-inflammatory microenvironment [19,20,21]. Furthermore, Piezo 1 has been implicated in extracellular matrix remodeling, fibrosis, and inflammatory mechanotransduction, establishing a feedback loop in which tissue stiffness and mechanical stress promote Piezo 1 activation, leading to calcium-dependent inflammatory signaling, cytokine production, and immune-cell activation. These inflammatory and remodeling processes may further alter extracellular matrix composition and increase tissue stiffness, thereby sustaining persistent Piezo 1 activation and perpetuating a pathological mechanoinflammatory cycle [17,18,19,20]. Increased tissue stiffness enhances Piezo 1 activation, which in turn promotes further inflammation and ECM remodeling, perpetuating a pathological cycle. Mechanosensitive signaling pathways have also been associated with nociceptive processing. Increased intracellular calcium levels can enhance neuronal excitability and facilitate pain transmission, contributing to peripheral and central sensitization [12,13].

These mechanisms support the hypothesis that Piezo 1 may contribute to pathways relevant to chronic pain. Mechanosensitive ion channels are increasingly recognized as regulators of neuronal excitability, immune activation, and persistent pain signaling, linking biomechanical stress to nociceptive responses [31].

Despite this strong biological rationale, our results indicate that circulating Piezo 1 levels do not correlate with clinical parameters. This discrepancy likely reflects the predominantly local nature of Piezo 1 activity. Recent evidence suggests that Piezo 1 signaling is highly context-dependent and strongly influenced by local mechanical cues, extracellular matrix stiffness, and cell-specific microenvironmental conditions [19,31,32]. This suggests that systemic Piezo 1 expression is not responsive to clinical improvement and reinforces the hypothesis that its activity is predominantly confined to local tissue microenvironments.

Mechanotransduction is a tissue-specific process occurring within the affected microenvironment and may not be adequately captured by systemic measurements. Piezo 1-mediated signaling has been shown to regulate macrophage polarization, innate immune responses, and local inflammatory cascades, supporting a predominantly site-restricted biological activity [19,31,33].

The longitudinal analysis revealed no significant changes in circulating Piezo 1 levels following treatment, with only minimal, non-significant variation over time. This lack of temporal modulation further supports the notion that Piezo 1 is not a dynamic systemic biomarker in CRPS-1, despite its mechanistic relevance at the tissue level. The modest association observed with time from treatment suggests that Piezo 1 may be influenced by long-term biological adaptations, potentially related to tissue remodeling or chronic inflammatory processes, as described in mechanically driven pathological settings [32,34,35]. Reduced weight-bearing, functional disuse, and altered musculoskeletal mechanobiology associated with chronic CRPS-1 may also contribute to dysregulated PIEZO1-related signaling and potentially to lower circulating PIEZO1 levels. However, this finding should be interpreted within the limitations of the study design. The observed association may also be influenced by heterogeneous clinical trajectories, disease duration, and recovery stage, rather than representing a direct temporal effect of neridronate treatment itself. Furthermore, the absence of significant correlations with clinical outcomes suggests that these variations are not directly linked to symptom improvement.

Similarly, correlations observed with selected hematological and biochemical parameters should be interpreted cautiously due to the exploratory nature of these analyses, the relatively limited sample size, and the absence of correction for multiple testing. Therefore, these findings should be considered hypothesis-generating and require confirmation in larger independent cohorts.

Importantly, these findings highlight the value of negative results in biomarker research. In complex conditions such as CRPS-1, biologically plausible molecules do not necessarily translate into clinically useful circulating markers. The identification of non-informative biomarkers is essential to refine research strategies and improve future biomarker selection frameworks [36].

Taken together, our findings suggest a possible association between altered circulating Piezo 1 levels and CRPS-1 status. Although previous experimental studies support a potential role of Piezo 1-mediated mechanotransduction in inflammatory and nociceptive pathways, the present data do not provide direct evidence of causal mechanisms or tissue-specific Piezo 1 activity in CRPS-1.

The absence of significant associations with clinical parameters suggests that circulating Piezo 1 may have limited utility as a systemic biomarker in CRPS-1.

Future studies should focus on tissue-specific analyses and functional investigations, particularly within affected peripheral tissues and immune compartments where Piezo 1 signaling may be most biologically relevant [19,31,32,33].

This study has several limitations that should be acknowledged. First, the relatively small sample size may have limited the statistical power of some exploratory analyses. Second, circulating Piezo 1 levels were measured in serum samples only, without direct assessment of tissue-specific expression or functional activity. In addition, differences in age and sex distribution between groups may represent potential confounding factors and should be considered when interpreting the findings. Therefore, no conclusions can be drawn regarding local mechanotransductive mechanisms in affected tissues. Third, the observational design of the study does not allow causal inferences regarding the relationship between Piezo 1 alterations and CRPS-1 pathophysiology. Finally, the longitudinal analysis was performed in a limited subgroup of patients and should therefore be considered exploratory.

## 5. Conclusions

Circulating Piezo 1 levels were significantly altered in CRPS-1 patients compared with healthy controls, supporting a potential association between mechanosensitive pathways and disease status. However, no significant correlations were observed with self-perceived health status or treatment response. These findings suggest that circulating Piezo 1 has limited utility as a clinical biomarker in CRPS-1. Further studies are needed to clarify the biological significance of Piezo 1 alterations and to investigate its potential role in inflammatory and mechanotransductive processes.

## Figures and Tables

**Table 1:** Demographic and clinical characteristics of the study population.

Variable	CRPS-1 (n = 31)	Controls (n = 22)
Age (Years)	63.7 ± 13.6	55.2 ± 15.4
Female, n (%)	21 (67.7)	10 (45.5)
Male, n (%)	10 (32.3)	12 (54.5)
Months from neridronate treatment	18.2 ± 20.7	
Vitamin D supplementation, n (%)	19 (61.3)	
Affected site		
foot and ankle, n (%)	23 (74.2)	
hand and wrist, n (%)	8 (25.8)	
Comorbidities		
Autoimmune inflammatory rheumatic diseases, n (%)	5 (16.1)	
Diabetes, n (%)	3 (9.7)	
Hypertension, n (%)	13 (41.9)	
Thyroid disease, n (%)	8 (25.8)	
History of cancer, n (%)	3 (9.7)	
Osteoporosis, n (%)	9 (29)	
Budapest criteria domains at diagnosis		
Sensory, n (%)	23 (74.)	
Vasomotor, n (%)	11 (35.5)	
Sudomotor/edema, n (%)	12 (38.7)	
Motor/trophic, n (%)	26 (83.9)	
Inciting event		
Fracture, n (%)	9 (29.0)	
Surgery, n (%):	6 (19.4)	
Fracture and surgery, n (%)	2 (6.5)	
Trauma without fracture, n (%)	5 (16.1)	
Mechanical overload, n (%)	2 (6.5)	
None identified, n (%)	7 (22.6)	

Data are presented as mean ± standard deviation or number (percentage).

## Data Availability

The raw data supporting the findings of this study have been deposited in the Zenodo repository and will be publicly available upon publication of the article.

## Supplementary Materials

The following supporting information can be downloaded at https://www.mdpi.com/article/10.3390/biomedicines14061266/s1. STROBE Checklist; STROBE flow chart.
