# Supplementary material for: Circulating Piezo 1 Levels in Complex Regional Pain Syndrome Type 1 and Their Association with Time from Neridronate Treatment
Source: Biomedicines. 2026 Jun 1;14(6):1266. doi: 10.3390/biomedicines14061266 (PMC13297369; doi:10.3390/biomedicines14061266)
Supplement: Supplementary file 1 [file biomedicines-14-01266-s001.zip › biomedicines-4308209-supplementary.pdf]

biomedicines

MDPI

SUPPLEMENTARY MATERIALS

Circulating PIEZO1 Levels in Complex Regional Pain Syndrome Type 1 and Their Association with Time from Neridronate Treatment

Elisa Assirelli [1], Jacopo Ciaffi [1,2] , Susanna Naldi[1], Francesco Ursini[1,2] and Simona Neri 1[,*]

1 Medicine and Rheumatology Unit, IRCCS Istituto Ortopedico Rizzoli, Bologna, Italy; elisa.assirelli@ior.it, jacopo.ciaffi@ior.it, susanna.naldi@ior.it; francesco.ursini@ior.it; simona.neri@ior.it
2 Department of Biomedical and Neuromotor Sciences (DIBINEM), Alma Mater Studiorum University of Bologna, Bologna, Italy;
*Correspondence: simona.neri@ior.it

 https://doi.org/10.3390/biomedicines14061266

STROBE CHECKLIST

**Section**	**Item**	**Recommendation**	**Page No.**
**Title and abstract**	1a	Exploratory observational case-control biomarker study titled "Circulating PIEZO1 Levels in Complex Regional Pain Syndrome Type 1 and Their Association with Time from Neridronate Treatment"	1
	1b	Abstract including background, methods, results and conclusions	1
**Introduction**	2	Scientific background and rationale	2–3
	3	Objectives: evaluate circulating PIEZO1 levels and associations with clinical parameters and treatment response	3
**Methods**	4	Study design: exploratory observational case-control biomarker study presented in Methods	3–4
	5	Setting, recruitment source, biobank origin, ethics approval, and sampling period described	4
	6	Eligibility criteria, participant selection described; CRPS-1 diagnosed according to Budapest Criteria; controls from Rheumatology Biobank	4
	7	Outcomes, exposures, predictors, and clinical variables defined	4–5
	8	ELISA assay methods, kit characteristics, serum handling and measurement procedures described	5
	9	Potential bias discussed in limitations section (small sample size, age/sex imbalance, serum-only measurements)	11–12
	10	Sample size not formally justified by power analysis (no reference effect size estimations available in the literature).	?
	11	Quantitative variables handled as continuous variables; descriptive statistics and correlations explained	5
	12a	Statistical methods described (Mann–Whitney, Wilcoxon, paired t-test, Spearman correlations)	5
	12b	Exploratory subgroup analyses described (paired pre/post-treatment subgroup; responders vs non-responders)	5–6
**Results**	13a	Numbers of participants included in CRPS-1 and control groups reported	5,6
	13b	Reasons for non-participation	N/A
	13c	Flow diagram for patient samples	Suppl.
	14a	Demographic and clinical characteristics summarized in Table 1	6
	14b	Missing data per variable not explicitly reported	Not reported
	14c	Follow-up time summarized through months from treatment	4, 6
	15	Outcome measures and summary statistics for PIEZO1 concentrations reported	6–8
	16a	Unadjusted estimates and p-values reported; no adjusted analyses performed	6–8
	16b	No categorized continuous variables requiring category boundaries	N/A
	16c	Relative risks not applicable	N/A
	17	Additional exploratory analyses with laboratory variables described	8
**Discussion**	18	Key results summarized with reference to objectives	8–12
	19	Limitations discussed, including sample size, observational design, serum-only analysis, confounding factors	11–12
	20	Interpretation balanced and cautious, acknowledging negative biomarker findings	8–12
	21	Generalisability indirectly discussed; further studies recommended	11–12
**Other information**	22	Funding source reported	13

https://doi.org/10.3390/biomedicines14061266

STROBE FLOW CHART

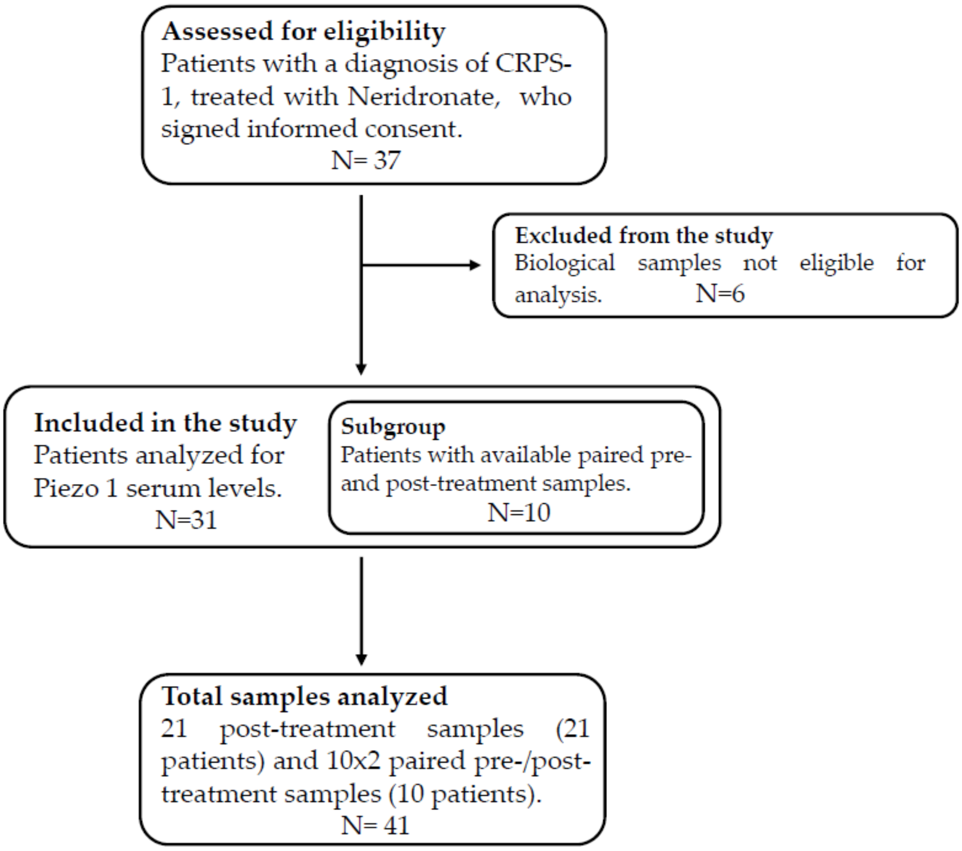

https://doi.org/10.3390/biomedicines14061266
